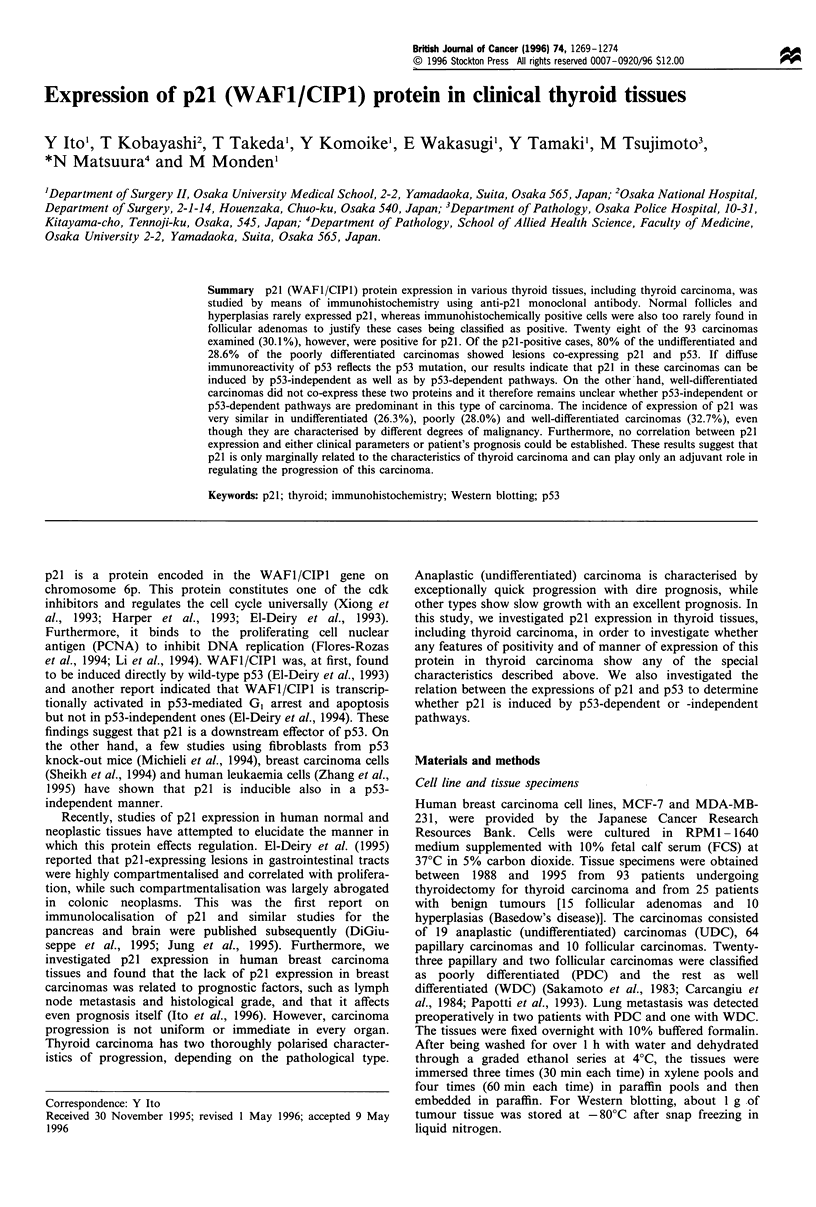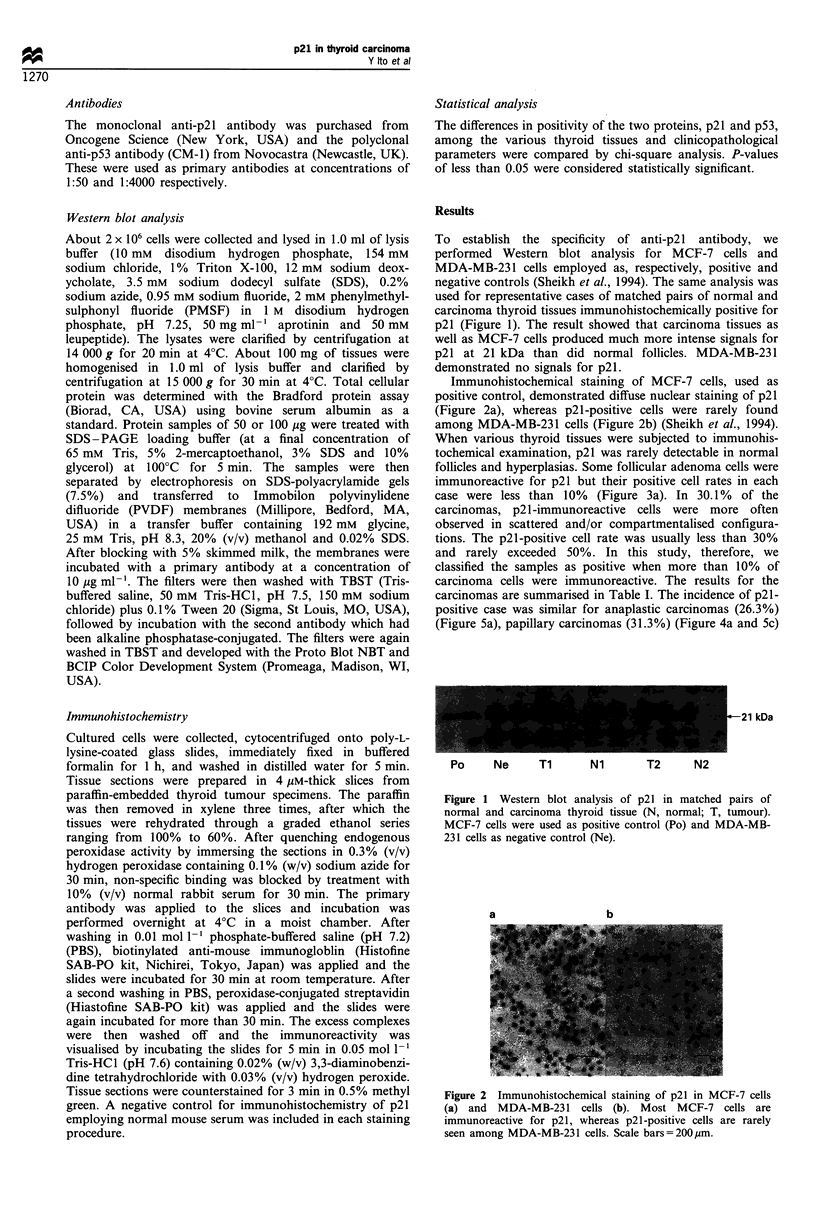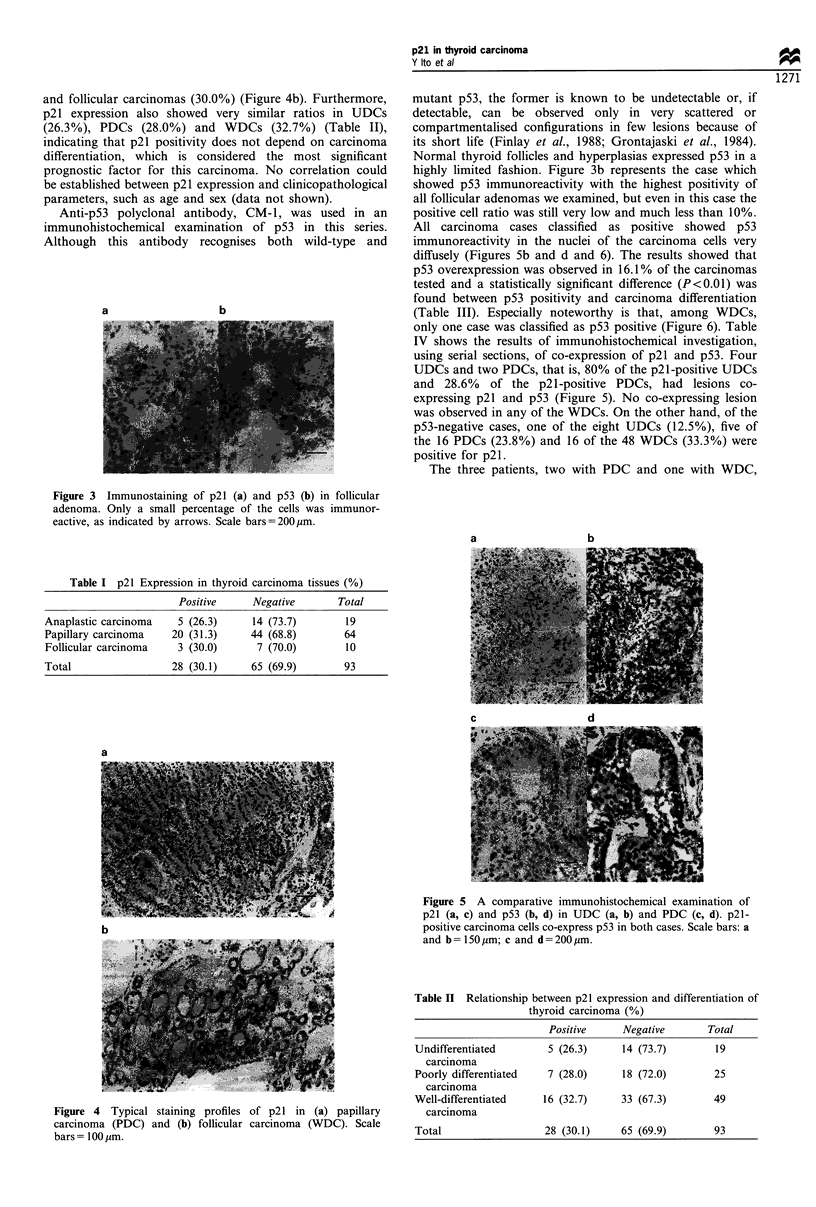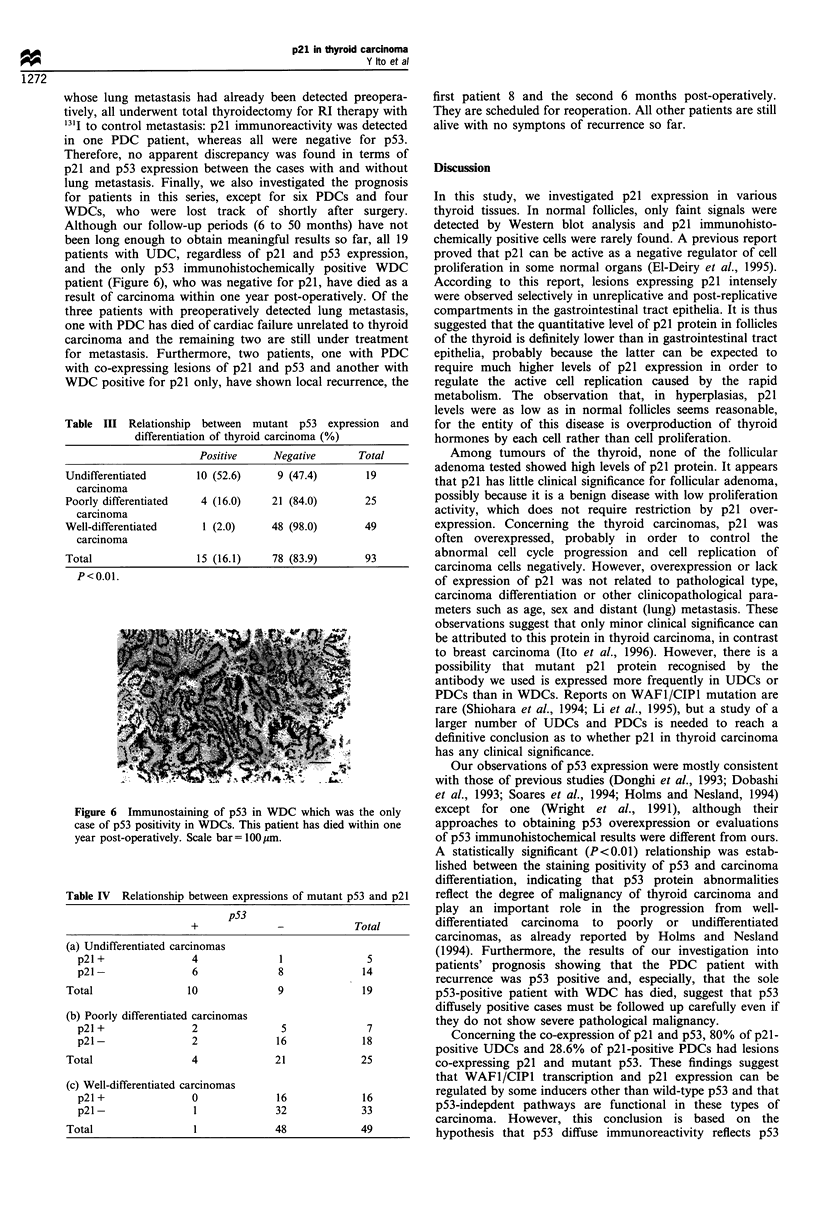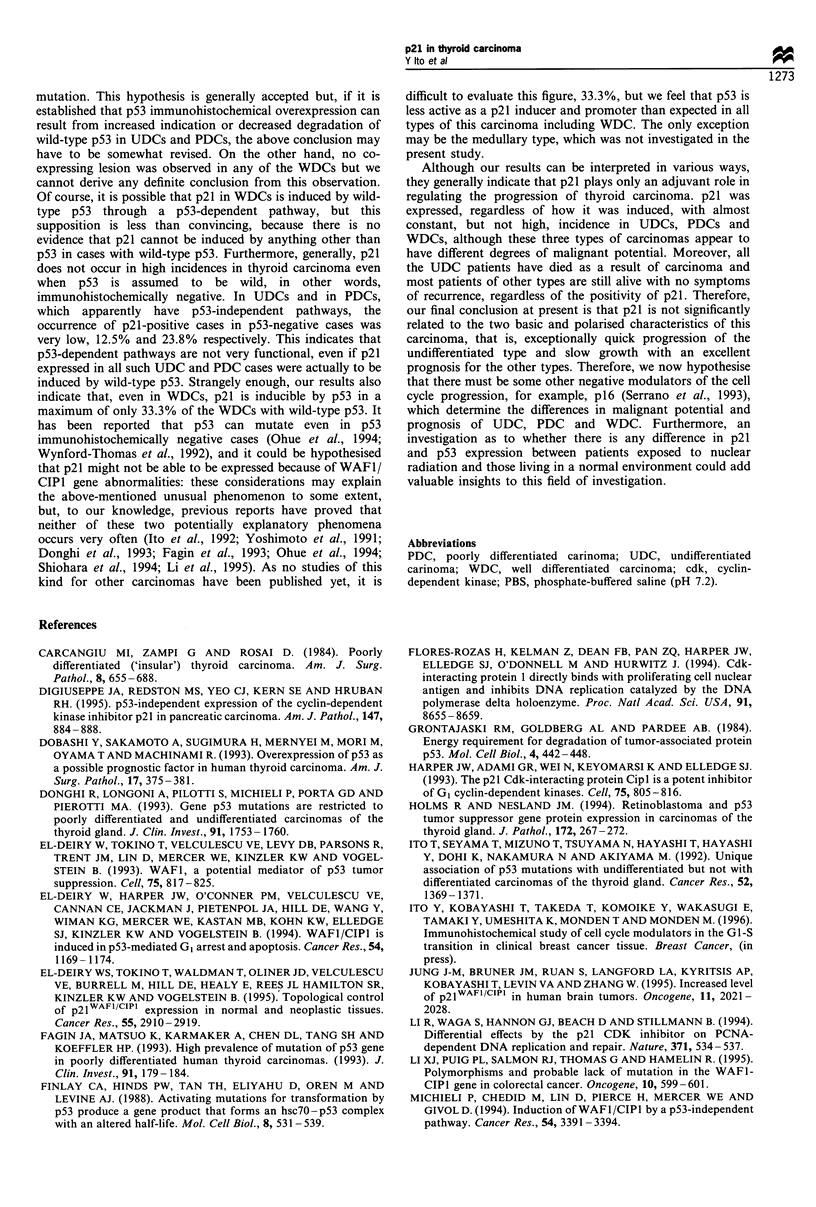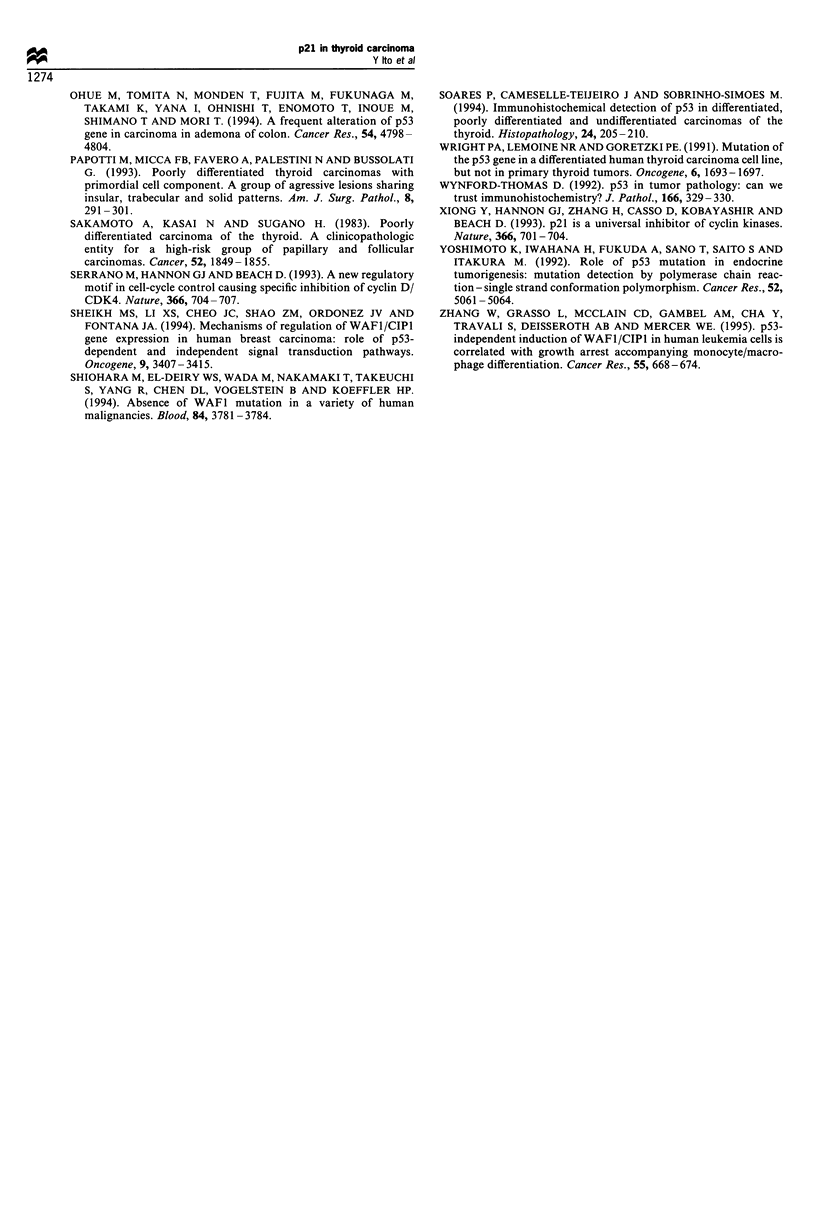# Expression of p21 (WAF1/CIP1) protein in clinical thyroid tissues.

**DOI:** 10.1038/bjc.1996.528

**Published:** 1996-10

**Authors:** Y. Ito, T. Kobayashi, T. Takeda, Y. Komoike, E. Wakasugi, Y. Tamaki, M. Tsujimoto, N. Matsuura, M. Monden

**Affiliations:** Department of Surgery II, Osaka University Medical School, Japan.

## Abstract

**Images:**


British Journal of Cancer (1996) 74, 1269-1274
© 1996 Stockton Press All rights reserved 0007-0920/96 $12.00

### Expression of p21 (WAF1/CIP1) protein in clinical thyroid tissues

Y Ito[1], T Kobayashi[2], T Takeda[1], Y Komoike[1], E Wakasugi[1], Y Tamaki[1], M Tsujimoto[3], *N Matsuura[4] and M Monden[1]

[1]*Department of Surgery II, Osaka University Medical School, 2-2, Yamadaoka, Suita, Osaka 565, Japan;* [2]*Osaka National Hospital, Department of Surgery, 2-1-14, Houenzaka, Chuo-ku, Osaka 540, Japan;* [3]*Department of Pathology, Osaka Police Hospital, 10-31, Kitayama-cho, Tennoji-ku, Osaka, 545, Japan;* [4]*Department of Pathology, School of Allied Health Science, Faculty of Medicine, Osaka University 2-2, Yamadaoka, Suita, Osaka 565, Japan.*

**Summary** p21 (WAF1/CIP1) protein expression in various thyroid tissues, including thyroid carcinoma, was studied by means of immunohistochemistry using anti-p21 monoclonal antibody. Normal follicles and hyperplasias rarely expressed p21, whereas immunohistochemically positive cells were also too rarely found in follicular adenomas to justify these cases being classified as positive. Twenty eight of the 93 carcinomas examined (30.1%), however, were positive for p21. Of the p21-positive cases, 80% of the undifferentiated and 28.6% of the poorly differentiated carcinomas showed lesions co-expressing p21 and p53. If diffuse immunoreactivity of p53 reflects the p53 mutation, our results indicate that p21 in these carcinomas can be induced by p53-independent as well as by p53-dependent pathways. On the other hand, well-differentiated carcinomas did not co-express these two proteins and it therefore remains unclear whether p53-independent or p53-dependent pathways are predominant in this type of carcinoma. The incidence of expression of p21 was very similar in undifferentiated (26.3%), poorly (28.0%) and well-differentiated carcinomas (32.7%), even though they are characterised by different degrees of malignancy. Furthermore, no correlation between p21 expression and either clinical parameters or patient's prognosis could be established. These results suggest that p21 is only marginally related to the characteristics of thyroid carcinoma and can play only an adjuvant role in regulating the progression of this carcinoma.

**Keywords:** p21; thyroid; immunohistochemistry; Western blotting; p53

p21 is a protein encoded in the WAF1/CIP1 gene on chromosome 6p. This protein constitutes one of the cdk inhibitors and regulates the cell cycle universally (Xiong *et al.*, 1993; Harper *et al.*, 1993; El-Deiry *et al.*, 1993). Furthermore, it binds to the proliferating cell nuclear antigen (PCNA) to inhibit DNA replication (Flores-Rozas *et al.*, 1994; Li *et al.*, 1994). WAF1/CIP1 was, at first, found to be induced directly by wild-type p53 (El-Deiry *et al.*, 1993) and another report indicated that WAF1/CIP1 is transcriptionally activated in p53-mediated $G_1$ arrest and apoptosis but not in p53-independent ones (El-Deiry *et al.*, 1994). These findings suggest that p21 is a downstream effector of p53. On the other hand, a few studies using fibroblasts from p53 knock-out mice (Michieli *et al.*, 1994), breast carcinoma cells (Sheikh *et al.*, 1994) and human leukaemia cells (Zhang *et al.*, 1995) have shown that p21 is inducible also in a p53-independent manner.

Recently, studies of p21 expression in human normal and neoplastic tissues have attempted to elucidate the manner in which this protein effects regulation. El-Deiry *et al.* (1995) reported that p21-expressing lesions in gastrointestinal tracts were highly compartmentalised and correlated with proliferation, while such compartmentalisation was largely abrogated in colonic neoplasms. This was the first report on immunolocalisation of p21 and similar studies for the pancreas and brain were published subsequently (DiGiuseppe *et al.*, 1995; Jung *et al.*, 1995). Furthermore, we investigated p21 expression in human breast carcinoma tissues and found that the lack of p21 expression in breast carcinomas was related to prognostic factors, such as lymph node metastasis and histological grade, and that it affects even prognosis itself (Ito *et al.*, 1996). However, carcinoma progression is not uniform or immediate in every organ. Thyroid carcinoma has two thoroughly polarised characteristics of progression, depending on the pathological type. Anaplastic (undifferentiated) carcinoma is characterised by exceptionally quick progression with dire prognosis, while other types show slow growth with an excellent prognosis. In this study, we investigated p21 expression in thyroid tissues, including thyroid carcinoma, in order to investigate whether any features of positivity and of manner of expression of this protein in thyroid carcinoma show any of the special characteristics described above. We also investigated the relation between the expressions of p21 and p53 to determine whether p21 is induced by p53-dependent or -independent pathways.

Correspondence: Y Ito
Received 30 November 1995; revised 1 May 1996; accepted 9 May 1996

### Materials and methods

### *Cell line and tissue specimens*

Human breast carcinoma cell lines, MCF-7 and MDA-MB-231, were provided by the Japanese Cancer Research Resources Bank. Cells were cultured in RPM1-1640 medium supplemented with 10% fetal calf serum (FCS) at 37°C in 5% carbon dioxide. Tissue specimens were obtained between 1988 and 1995 from 93 patients undergoing thyroidectomy for thyroid carcinoma and from 25 patients with benign tumours [15 follicular adenomas and 10 hyperplasias (Basedow's disease)]. The carcinomas consisted of 19 anaplastic (undifferentiated) carcinomas (UDC), 64 papillary carcinomas and 10 follicular carcinomas. Twenty-three papillary and two follicular carcinomas were classified as poorly differentiated (PDC) and the rest as well differentiated (WDC) (Sakamoto *et al.*, 1983; Carcangiu *et al.*, 1984; Papotti *et al.*, 1993). Lung metastasis was detected preoperatively in two patients with PDC and one with WDC. The tissues were fixed overnight with 10% buffered formalin. After being washed for over 1 h with water and dehydrated through a graded ethanol series at 4°C, the tissues were immersed three times (30 min each time) in xylene pools and four times (60 min each time) in paraffin pools and then embedded in paraffin. For Western blotting, about 1 g of tumour tissue was stored at −80°C after snap freezing in liquid nitrogen.

*Antibodies*

The monoclonal anti-p21 antibody was purchased from Oncogene Science (New York, USA) and the polyclonal anti-p53 antibody (CM-1) from Novocastra (Newcastle, UK). These were used as primary antibodies at concentrations of 1:50 and 1:4000 respectively.

*Western blot analysis*

About $2\times10^6$ cells were collected and lysed in 1.0 ml of lysis buffer (10 mM disodium hydrogen phosphate, 154 mM sodium chloride, 1% Triton X-100, 12 mM sodium deoxycholate, 3.5 mM sodium dodecyl sulfate (SDS), 0.2% sodium azide, 0.95 mM sodium fluoride, 2 mM phenylmethylsulphonyl fluoride (PMSF) in 1 M disodium hydrogen phosphate, pH 7.25, 50 mg ml$^{-1}$ aprotinin and 50 mM leupeptide). The lysates were clarified by centrifugation at 14 000 ***g*** for 20 min at 4°C. About 100 mg of tissues were homogenised in 1.0 ml of lysis buffer and clarified by centrifugation at 15 000 ***g*** for 30 min at 4°C. Total cellular protein was determined with the Bradford protein assay (Biorad, CA, USA) using bovine serum albumin as a standard. Protein samples of 50 or 100 $\mu$g were treated with SDS–PAGE loading buffer (at a final concentration of 65 mM Tris, 5% 2-mercaptoethanol, 3% SDS and 10% glycerol) at 100°C for 5 min. The samples were then separated by electrophoresis on SDS-polyacrylamide gels (7.5%) and transferred to Immobilon polyvinylidene difluoride (PVDF) membranes (Millipore, Bedford, MA, USA) in a transfer buffer containing 192 mM glycine, 25 mM Tris, pH 8.3, 20% (v/v) methanol and 0.02% SDS. After blocking with 5% skimmed milk, the membranes were incubated with a primary antibody at a concentration of 10 $\mu$g ml$^{-1}$. The filters were then washed with TBST (Tris-buffered saline, 50 mM Tris-HCl, pH 7.5, 150 mM sodium chloride) plus 0.1% Tween 20 (Sigma, St Louis, MO, USA), followed by incubation with the second antibody which had been alkaline phosphatase-conjugated. The filters were again washed in TBST and developed with the Proto Blot NBT and BCIP Color Development System (Promeaga, Madison, WI, USA).

*Immunohistochemistry*

Cultured cells were collected, cytocentrifuged onto poly-L-lysine-coated glass slides, immediately fixed in buffered formalin for 1 h, and washed in distilled water for 5 min. Tissue sections were prepared in 4 $\mu$M-thick slices from paraffin-embedded thyroid tumour specimens. The paraffin was then removed in xylene three times, after which the tissues were rehydrated through a graded ethanol series ranging from 100% to 60%. After quenching endogenous peroxidase activity by immersing the sections in 0.3% (v/v) hydrogen peroxidase containing 0.1% (w/v) sodium azide for 30 min, non-specific binding was blocked by treatment with 10% (v/v) normal rabbit serum for 30 min. The primary antibody was applied to the slices and incubation was performed overnight at 4°C in a moist chamber. After washing in 0.01 mol l$^{-1}$ phosphate-buffered saline (pH 7.2) (PBS), biotinylated anti-mouse immunogloblin (Histofine SAB-PO kit, Nichirei, Tokyo, Japan) was applied and the slides were incubated for 30 min at room temperature. After a second washing in PBS, peroxidase-conjugated streptavidin (Hiastofine SAB-PO kit) was applied and the slides were again incubated for more than 30 min. The excess complexes were then washed off and the immunoreactivity was visualised by incubating the slides for 5 min in 0.05 mol l$^{-1}$ Tris-HCl (pH 7.6) containing 0.02% (w/v) 3,3-diaminobenzidine tetrahydrochloride with 0.03% (v/v) hydrogen peroxide. Tissue sections were counterstained for 3 min in 0.5% methyl green. A negative control for immunohistochemistry of p21 employing normal mouse serum was included in each staining procedure.

*Statistical analysis*

The differences in positivity of the two proteins, p21 and p53, among the various thyroid tissues and clinicopathological parameters were compared by chi-square analysis. *P*-values of less than 0.05 were considered statistically significant.

### Results

To establish the specificity of anti-p21 antibody, we performed Western blot analysis for MCF-7 cells and MDA-MB-231 cells employed as, respectively, positive and negative controls (Sheikh *et al.*, 1994). The same analysis was used for representative cases of matched pairs of normal and carcinoma thyroid tissues immunohistochemically positive for p21 (Figure 1). The result showed that carcinoma tissues as well as MCF-7 cells produced much more intense signals for p21 at 21 kDa than did normal follicles. MDA-MB-231 demonstrated no signals for p21.

Immunohistochemical staining of MCF-7 cells, used as positive control, demonstrated diffuse nuclear staining of p21 (Figure 2a), whereas p21-positive cells were rarely found among MDA-MB-231 cells (Figure 2b) (Sheikh *et al.*, 1994). When various thyroid tissues were subjected to immunohistochemical examination, p21 was rarely detectable in normal follicles and hyperplasias. Some follicular adenoma cells were immunoreactive for p21 but their positive cell rates in each case were less than 10% (Figure 3a). In 30.1% of the carcinomas, p21-immunoreactive cells were more often observed in scattered and/or compartmentalised configurations. The p21-positive cell rate was usually less than 30% and rarely exceeded 50%. In this study, therefore, we classified the samples as positive when more than 10% of carcinoma cells were immunoreactive. The results for the carcinomas are summarised in Table I. The incidence of p21-positive case was similar for anaplastic carcinomas (26.3%) (Figure 5a), papillary carcinomas (31.3%) (Figure 4a and 5c)

**Figure 1** Western blot analysis of p21 in matched pairs of normal and carcinoma thyroid tissue (N, normal; T, tumour). MCF-7 cells were used as positive control (Po) and MDA-MB-231 cells as negative control (Ne).

**Figure 2** Immunohistochemical staining of p21 in MCF-7 cells (**a**) and MDA-MB-231 cells (**b**). Most MCF-7 cells are immunoreactive for p21, whereas p21-positive cells are rarely seen among MDA-MB-231 cells. Scale bars = 200 $\mu$m.

and follicular carcinomas (30.0%) (Figure 4b). Furthermore, p21 expression also showed very similar ratios in UDCs (26.3%), PDCs (28.0%) and WDCs (32.7%) (Table II), indicating that p21 positivity does not depend on carcinoma differentiation, which is considered the most significant prognostic factor for this carcinoma. No correlation could be established between p21 expression and clinicopathological parameters, such as age and sex (data not shown).

Anti-p53 polyclonal antibody, CM-1, was used in an immunohistochemical examination of p53 in this series. Although this antibody recognises both wild-type and mutant p53, the former is known to be undetectable or, if detectable, can be observed only in very scattered or compartmentalised configurations in few lesions because of its short life (Finlay *et al.*, 1988; Grontajaski *et al.*, 1984). Normal thyroid follicles and hyperplasias expressed p53 in a highly limited fashion. Figure 3b represents the case which showed p53 immunoreactivity with the highest positivity of all follicular adenomas we examined, but even in this case the positive cell ratio was still very low and much less than 10%. All carcinoma cases classified as positive showed p53 immunoreactivity in the nuclei of the carcinoma cells very diffusely (Figures 5b and d and 6). The results showed that p53 overexpression was observed in 16.1% of the carcinomas tested and a statistically significant difference ($P<0.01$) was found between p53 positivity and carcinoma differentiation (Table III). Especially noteworthy is that, among WDCs, only one case was classified as p53 positive (Figure 6). Table IV shows the results of immunohistochemical investigation, using serial sections, of co-expression of p21 and p53. Four UDCs and two PDCs, that is, 80% of the p21-positive UDCs and 28.6% of the p21-positive PDCs, had lesions co-expressing p21 and p53 (Figure 5). No co-expressing lesion was observed in any of the WDCs. On the other hand, of the p53-negative cases, one of the eight UDCs (12.5%), five of the 16 PDCs (23.8%) and 16 of the 48 WDCs (33.3%) were positive for p21.

The three patients, two with PDC and one with WDC,

**Figure 3** Immunostaining of p21 (**a**) and p53 (**b**) in follicular adenoma. Only a small percentage of the cells was immunoreactive, as indicated by arrows. Scale bars = 200 $\mu$m.

**Table I** p21 Expression in thyroid carcinoma tissues (%)

| | *Positive* | *Negative* | *Total* |
|---|---|---|---|
| Anaplastic carcinoma | 5 (26.3) | 14 (73.7) | 19 |
| Papillary carcinoma | 20 (31.3) | 44 (68.8) | 64 |
| Follicular carcinoma | 3 (30.0) | 7 (70.0) | 10 |
| Total | 28 (30.1) | 65 (69.9) | 93 |

**Figure 4** Typical staining profiles of p21 in (**a**) papillary carcinoma (PDC) and (**b**) follicular carcinoma (WDC). Scale bars = 100 $\mu$m.

**Figure 5** A comparative immunohistochemical examination of p21 (**a, c**) and p53 (**b, d**) in UDC (**a, b**) and PDC (**c, d**). p21-positive carcinoma cells co-express p53 in both cases. Scale bars: **a** and **b** = 150 $\mu$m; **c** and **d** = 200 $\mu$m.

**Table II** Relationship between p21 expression and differentiation of thyroid carcinoma (%)

| | *Positive* | *Negative* | *Total* |
|---|---|---|---|
| Undifferentiated carcinoma | 5 (26.3) | 14 (73.7) | 19 |
| Poorly differentiated carcinoma | 7 (28.0) | 18 (72.0) | 25 |
| Well-differentiated carcinoma | 16 (32.7) | 33 (67.3) | 49 |
| Total | 28 (30.1) | 65 (69.9) | 93 |

whose lung metastasis had already been detected preoperatively, all underwent total thyroidectomy for RI therapy with $^{131}I$ to control metastasis: p21 immunoreactivity was detected in one PDC patient, whereas all were negative for p53. Therefore, no apparent discrepancy was found in terms of p21 and p53 expression between the cases with and without lung metastasis. Finally, we also investigated the prognosis for patients in this series, except for six PDCs and four WDCs, who were lost track of shortly after surgery. Although our follow-up periods (6 to 50 months) have not been long enough to obtain meaningful results so far, all 19 patients with UDC, regardless of p21 and p53 expression, and the only p53 immunohistochemically positive WDC patient (Figure 6), who was negative for p21, have died as a result of carcinoma within one year post-operatively. Of the three patients with preoperatively detected lung metastasis, one with PDC has died of cardiac failure unrelated to thyroid carcinoma and the remaining two are still under treatment for metastasis. Furthermore, two patients, one with PDC with co-expressing lesions of p21 and p53 and another with WDC positive for p21 only, have shown local recurrence, the first patient 8 and the second 6 months post-operatively. They are scheduled for reoperation. All other patients are still alive with no symptons of recurrence so far.

**Table III** Relationship between mutant p53 expression and differentiation of thyroid carcinoma (%)

| | *Positive* | *Negative* | *Total* |
|---|---|---|---|
| Undifferentiated carcinoma | 10 (52.6) | 9 (47.4) | 19 |
| Poorly differentiated carcinoma | 4 (16.0) | 21 (84.0) | 25 |
| Well-differentiated carcinoma | 1 (2.0) | 48 (98.0) | 49 |
| Total | 15 (16.1) | 78 (83.9) | 93 |

$P < 0.01$.

**Figure 6** Immunostaining of p53 in WDC which was the only case of p53 positivity in WDCs. This patient has died within one year post-operatively. Scale bar = 100 $\mu$m.

**Table IV** Relationship between expressions of mutant p53 and p21

| | *p53* + | *p53* − | *Total* |
|---|---|---|---|
| (a) Undifferentiated carcinomas | | | |
| p21+ | 4 | 1 | 5 |
| p21− | 6 | 8 | 14 |
| Total | 10 | 9 | 19 |
| (b) Poorly differentiated carcinomas | | | |
| p21+ | 2 | 5 | 7 |
| p21− | 2 | 16 | 18 |
| Total | 4 | 21 | 25 |
| (c) Well-differentiated carcinomas | | | |
| p21+ | 0 | 16 | 16 |
| p21− | 1 | 32 | 33 |
| Total | 1 | 48 | 49 |

### Discussion

In this study, we investigated p21 expression in various thyroid tissues. In normal follicles, only faint signals were detected by Western blot analysis and p21 immunohistochemically positive cells were rarely found. A previous report proved that p21 can be active as a negative regulator of cell proliferation in some normal organs (El-Deiry *et al.*, 1995). According to this report, lesions expressing p21 intensely were observed selectively in unreplicative and post-replicative compartments in the gastrointestinal tract epithelia. It is thus suggested that the quantitative level of p21 protein in follicles of the thyroid is definitely lower than in gastrointestinal tract epithelia, probably because the latter can be expected to require much higher levels of p21 expression in order to regulate the active cell replication caused by the rapid metabolism. The observation that, in hyperplasias, p21 levels were as low as in normal follicles seems reasonable, for the entity of this disease is overproduction of thyroid hormones by each cell rather than cell proliferation.

Among tumours of the thyroid, none of the follicular adenoma tested showed high levels of p21 protein. It appears that p21 has little clinical significance for follicular adenoma, possibly because it is a benign disease with low proliferation activity, which does not require restriction by p21 overexpression. Concerning the thyroid carcinomas, p21 was often overexpressed, probably in order to control the abnormal cell cycle progression and cell replication of carcinoma cells negatively. However, overexpression or lack of expression of p21 was not related to pathological type, carcinoma differentiation or other clinicopathological parameters such as age, sex and distant (lung) metastasis. These observations suggest that only minor clinical significance can be attributed to this protein in thyroid carcinoma, in contrast to breast carcinoma (Ito *et al.*, 1996). However, there is a possibility that mutant p21 protein recognised by the antibody we used is expressed more frequently in UDCs or PDCs than in WDCs. Reports on WAF1/CIP1 mutation are rare (Shiohara *et al.*, 1994; Li *et al.*, 1995), but a study of a larger number of UDCs and PDCs is needed to reach a definitive conclusion as to whether p21 in thyroid carcinoma has any clinical significance.

Our observations of p53 expression were mostly consistent with those of previous studies (Donghi *et al.*, 1993; Dobashi *et al.*, 1993; Soares *et al.*, 1994; Holms and Nesland, 1994) except for one (Wright *et al.*, 1991), although their approaches to obtaining p53 overexpression or evaluations of p53 immunohistochemical results were different from ours. A statistically significant ($P < 0.01$) relationship was established between the staining positivity of p53 and carcinoma differentiation, indicating that p53 protein abnormalities reflect the degree of malignancy of thyroid carcinoma and play an important role in the progression from well-differentiated carcinoma to poorly or undifferentiated carcinomas, as already reported by Holms and Nesland (1994). Furthermore, the results of our investigation into patients' prognosis showing that the PDC patient with recurrence was p53 positive and, especially, that the sole p53-positive patient with WDC has died, suggest that p53 diffusely positive cases must be followed up carefully even if they do not show severe pathological malignancy.

Concerning the co-expression of p21 and p53, 80% of p21-positive UDCs and 28.6% of p21-positive PDCs had lesions co-expressing p21 and mutant p53. These findings suggest that WAF1/CIP1 transcription and p21 expression can be regulated by some inducers other than wild-type p53 and that p53-indepdent pathways are functional in these types of carcinoma. However, this conclusion is based on the hypothesis that p53 diffuse immunoreactivity reflects p53

mutation. This hypothesis is generally accepted but, if it is established that p53 immunohistochemical overexpression can result from increased indication or decreased degradation of wild-type p53 in UDCs and PDCs, the above conclusion may have to be somewhat revised. On the other hand, no co-expressing lesion was observed in any of the WDCs but we cannot derive any definite conclusion from this observation. Of course, it is possible that p21 in WDCs is induced by wild-type p53 through a p53-dependent pathway, but this supposition is less than convincing, because there is no evidence that p21 cannot be induced by anything other than p53 in cases with wild-type p53. Furthermore, generally, p21 does not occur in high incidences in thyroid carcinoma even when p53 is assumed to be wild, in other words, immunohistochemically negative. In UDCs and in PDCs, which apparently have p53-independent pathways, the occurrence of p21-positive cases in p53-negative cases was very low, 12.5% and 23.8% respectively. This indicates that p53-dependent pathways are not very functional, even if p21 expressed in all such UDC and PDC cases were actually to be induced by wild-type p53. Strangely enough, our results also indicate that, even in WDCs, p21 is inducible by p53 in a maximum of only 33.3% of the WDCs with wild-type p53. It has been reported that p53 can mutate even in p53 immunohistochemically negative cases (Ohue *et al.*, 1994; Wynford-Thomas *et al.*, 1992), and it could be hypothesised that p21 might not be able to be expressed because of WAF1/CIP1 gene abnormalities: these considerations may explain the above-mentioned unusual phenomenon to some extent, but, to our knowledge, previous reports have proved that neither of these two potentially explanatory phenomena occurs very often (Ito *et al.*, 1992; Yoshimoto *et al.*, 1991; Donghi *et al.*, 1993; Fagin *et al.*, 1993; Ohue *et al.*, 1994; Shiohara *et al.*, 1994; Li *et al.*, 1995). As no studies of this kind for other carcinomas have been published yet, it is difficult to evaluate this figure, 33.3%, but we feel that p53 is less active as a p21 inducer and promoter than expected in all types of this carcinoma including WDC. The only exception may be the medullary type, which was not investigated in the present study.

Although our results can be interpreted in various ways, they generally indicate that p21 plays only an adjuvant role in regulating the progression of thyroid carcinoma. p21 was expressed, regardless of how it was induced, with almost constant, but not high, incidence in UDCs, PDCs and WDCs, although these three types of carcinomas appear to have different degrees of malignant potential. Moreover, all the UDC patients have died as a result of carcinoma and most patients of other types are still alive with no symptoms of recurrence, regardless of the positivity of p21. Therefore, our final conclusion at present is that p21 is not significantly related to the two basic and polarised characteristics of this carcinoma, that is, exceptionally quick progression of the undifferentiated type and slow growth with an excellent prognosis for the other types. Therefore, we now hypothesise that there must be some other negative modulators of the cell cycle progression, for example, p16 (Serrano *et al.*, 1993), which determine the differences in malignant potential and prognosis of UDC, PDC and WDC. Furthermore, an investigation as to whether there is any difference in p21 and p53 expression between patients exposed to nuclear radiation and those living in a normal environment could add valuable insights to this field of investigation.

**Abbreviations**

PDC, poorly differentiated carinoma; UDC, undifferentiated carinoma; WDC, well differentiated carcinoma; cdk, cyclin-dependent kinase; PBS, phosphate-buffered saline (pH 7.2).

### References

CARCANGIU MI, ZAMPI G AND ROSAI D. (1984). Poorly differentiated ('insular') thyroid carcinoma. *Am. J. Surg. Pathol.*, **8,** 655–688.

DIGIUSEPPE JA, REDSTON MS, YEO CJ, KERN SE AND HRUBAN RH. (1995). p53-independent expression of the cyclin-dependent kinase inhibitor p21 in pancreatic carcinoma. *Am. J. Pathol.*, **147,** 884–888.

DOBASHI Y, SAKAMOTO A, SUGIMURA H, MERNYEI M, MORI M, OYAMA T AND MACHINAMI R. (1993). Overexpression of p53 as a possible prognostic factor in human thyroid carcinoma. *Am. J. Surg. Pathol.*, **17,** 375–381.

DONGHI R, LONGONI A, PILOTTI S, MICHIELI P, PORTA GD AND PIEROTTI MA. (1993). Gene p53 mutations are restricted to poorly differentiated and undifferentiated carcinomas of the thyroid gland. *J. Clin. Invest.*, **91,** 1753–1760.

EL-DEIRY W, TOKINO T, VELCULESCU VE, LEVY DB, PARSONS R, TRENT JM, LIN D, MERCER WE, KINZLER KW AND VOGELSTEIN B. (1993). WAF1, a potential mediator of p53 tumor suppression. *Cell*, **75,** 817–825.

EL-DEIRY W, HARPER JW, O'CONNER PM, VELCULESCU VE, CANNAN CE, JACKMAN J, PIETENPOL JA, HILL DE, WANG Y, WIMAN KG, MERCER WE, KASTAN MB, KOHN KW, ELLEDGE SJ, KINZLER KW AND VOGELSTEIN B. (1994). WAF1/CIP1 is induced in p53-mediated $G_1$ arrest and apoptosis. *Cancer Res.*, **54,** 1169–1174.

EL-DEIRY WS, TOKINO T, WALDMAN T, OLINER JD, VELCULESCU VE, BURRELL M, HILL DE, HEALY E, REES JL HAMILTON SR, KINZLER KW AND VOGELSTEIN B. (1995). Topological control of $p21^{WAF1/CIP1}$ expression in normal and neoplastic tissues. *Cancer Res.*, **55,** 2910–2919.

FAGIN JA, MATSUO K, KARMAKER A, CHEN DL, TANG SH AND KOEFFLER HP. (1993). High prevalence of mutation of p53 gene in poorly differentiated human thyroid carcinomas. (1993). *J. Clin. Invest.*, **91,** 179–184.

FINLAY CA, HINDS PW, TAN TH, ELIYAHU D, OREN M AND LEVINE AJ. (1988). Activating mutations for transformation by p53 produce a gene product that forms an hsc70–p53 complex with an altered half-life. *Mol. Cell Biol.*, **8,** 531–539.

FLORES-ROZAS H, KELMAN Z, DEAN FB, PAN ZQ, HARPER JW, ELLEDGE SJ, O'DONNELL M AND HURWITZ J. (1994). Cdk-interacting protein 1 directly binds with proliferating cell nuclear antigen and inhibits DNA replication catalyzed by the DNA polymerase delta holoenzyme. *Proc. Natl Acad. Sci. USA*, **91,** 8655–8659.

GRONTAJASKI RM, GOLDBERG AL AND PARDEE AB. (1984). Energy requirement for degradation of tumor-associated protein p53. *Mol. Cell Biol.*, **4,** 442–448.

HARPER JW, ADAMI GR, WEI N, KEYOMARSI K AND ELLEDGE SJ. (1993). The p21 Cdk-interacting protein Cip1 is a potent inhibitor of $G_1$ cyclin-dependent kinases. *Cell*, **75,** 805–816.

HOLMS R AND NESLAND JM. (1994). Retinoblastoma and p53 tumor suppressor gene protein expression in carcinomas of the thyroid gland. *J. Pathol.*, **172,** 267–272.

ITO T, SEYAMA T, MIZUNO T, TSUYAMA N, HAYASHI T, HAYASHI Y, DOHI K, NAKAMURA N AND AKIYAMA M. (1992). Unique association of p53 mutations with undifferentiated but not with differentiated carcinomas of the thyroid gland. *Cancer Res.*, **52,** 1369–1371.

ITO Y, KOBAYASHI T, TAKEDA T, KOMOIKE Y, WAKASUGI E, TAMAKI Y, UMESHITA K, MONDEN T AND MONDEN M. (1996). Immunohistochemical study of cell cycle modulators in the G1-S transition in clinical breast cancer tissue. *Breast Cancer*, (in press).

JUNG J-M, BRUNER JM, RUAN S, LANGFORD LA, KYRITSIS AP, KOBAYASHI T, LEVIN VA AND ZHANG W. (1995). Increased level of $p21^{WAF1/CIP1}$ in human brain tumors. *Oncogene*, **11,** 2021–2028.

LI R, WAGA S, HANNON GJ, BEACH D AND STILLMANN B. (1994). Differential effects by the p21 CDK inhibitor on PCNA-dependent DNA replication and repair. *Nature*, **371,** 534–537.

LI XJ, PUIG PL, SALMON RJ, THOMAS G AND HAMELIN R. (1995). Polymorphisms and probable lack of mutation in the WAF1-CIP1 gene in colorectal cancer. *Oncogene*, **10,** 599–601.

MICHIELI P, CHEDID M, LIN D, PIERCE H, MERCER WE AND GIVOL D. (1994). Induction of WAF1/CIP1 by a p53-independent pathway. *Cancer Res.*, **54,** 3391–3394.

OHUE M, TOMITA N, MONDEN T, FUJITA M, FUKUNAGA M, TAKAMI K, YANA I, OHNISHI T, ENOMOTO T, INOUE M, SHIMANO T AND MORI T. (1994). A frequent alteration of p53 gene in carcinoma in ademona of colon. *Cancer Res.*, **54**, 4798–4804.

PAPOTTI M, MICCA FB, FAVERO A, PALESTINI N AND BUSSOLATI G. (1993). Poorly differentiated thyroid carcinomas with primordial cell component. A group of agressive lesions sharing insular, trabecular and solid patterns. *Am. J. Surg. Pathol.*, **8**, 291–301.

SAKAMOTO A, KASAI N AND SUGANO H. (1983). Poorly differentiated carcinoma of the thyroid. A clinicopathologic entity for a high-risk group of papillary and follicular carcinomas. *Cancer*, **52**, 1849–1855.

SERRANO M, HANNON GJ AND BEACH D. (1993). A new regulatory motif in cell-cycle control causing specific inhibition of cyclin D/CDK4. *Nature*, **366**, 704–707.

SHEIKH MS, LI XS, CHEO JC, SHAO ZM, ORDONEZ JV AND FONTANA JA. (1994). Mechanisms of regulation of WAF1/CIP1 gene expression in human breast carcinoma: role of p53-dependent and independent signal transduction pathways. *Oncogene*, **9**, 3407–3415.

SHIOHARA M, EL-DEIRY WS, WADA M, NAKAMAKI T, TAKEUCHI S, YANG R, CHEN DL, VOGELSTEIN B AND KOEFFLER HP. (1994). Absence of WAF1 mutation in a variety of human malignancies. *Blood*, **84**, 3781–3784.

SOARES P, CAMESELLE-TEIJEIRO J AND SOBRINHO-SIMOES M. (1994). Immunohistochemical detection of p53 in differentiated, poorly differentiated and undifferentiated carcinomas of the thyroid. *Histopathology*, **24**, 205–210.

WRIGHT PA, LEMOINE NR AND GORETZKI PE. (1991). Mutation of the p53 gene in a differentiated human thyroid carcinoma cell line, but not in primary thyroid tumors. *Oncogene*, **6**, 1693–1697.

WYNFORD-THOMAS D. (1992). p53 in tumor pathology: can we trust immunohistochemistry? *J. Pathol.*, **166**, 329–330.

XIONG Y, HANNON GJ, ZHANG H, CASSO D, KOBAYASHIR AND BEACH D. (1993). p21 is a universal inhibitor of cyclin kinases. *Nature*, **366**, 701–704.

YOSHIMOTO K, IWAHANA H, FUKUDA A, SANO T, SAITO S AND ITAKURA M. (1992). Role of p53 mutation in endocrine tumorigenesis: mutation detection by polymerase chain reaction–single strand conformation polymorphism. *Cancer Res.*, **52**, 5061–5064.

ZHANG W, GRASSO L, MCCLAIN CD, GAMBEL AM, CHA Y, TRAVALI S, DEISSEROTH AB AND MERCER WE. (1995). p53-independent induction of WAF1/CIP1 in human leukemia cells is correlated with growth arrest accompanying monocyte/macrophage differentiation. *Cancer Res.*, **55**, 668–674.